# Do Early-Life Conditions Drive Variation in Senescence of Female Bighorn Sheep?

**DOI:** 10.3389/fcell.2021.637692

**Published:** 2021-05-20

**Authors:** Gabriel Pigeon, Julie Landes, Marco Festa-Bianchet, Fanie Pelletier

**Affiliations:** ^1^Département de Biologie, Université de Sherbrooke, Sherbrooke, QC, Canada; ^2^Faculty of Environmental Science and Nature Management, Norwegian University of Life Sciences, Ås, Norway; ^3^Centre de Recherche sur le Vieillissement, Sherbrooke, QC, Canada

**Keywords:** early-life, senescence, environmental conditions, long-term effects, life history, mortality

## Abstract

The rate of senescence may vary among individuals of a species according to individual life histories and environmental conditions. According to the principle of allocation, changes in mortality driven by environmental conditions influence how organisms allocate resources among costly functions. In several vertebrates, environmental conditions during early life impose trade-offs in allocation between early reproduction and maintenance. The effects of conditions experienced during early life on senescence, however, remain poorly documented in wild populations. We examined how several early-life environmental conditions affected reproductive and survival senescence in wild bighorn sheep. We found long-term effects of high population density at birth, precipitations during the winter before birth, and temperature during the winter following birth that decreased survival after 7 years of age. High temperature during the first summer and autumn of life and high Pacific decadal oscillation decreased reproductive success at old ages. However, harsh early-life environment did not influence the rate of senescence in either survival or reproduction. Contrary to our expectation, we found no trade-off between reproductive allocation prior to senescence and senescence. Our results do show that early-life environmental conditions are important drivers of later survival and reproductive success and contribute to intra-specific variation in late-life fitness, but not aging patterns. These conditions should therefore be considered when studying the mechanisms of senescence and the determinants of variation in both survival and reproductive senescence at older ages.

## Introduction

Demographic senescence is a decrease in survival and reproductive performance with age due to progressive decline in physiological functions (Kirkwood and Austad, 2000). The rate of senescence varies between species (Jones et al., 2014) but also among individuals of the same population. According to the disposable soma theory, senescence occurs because of trade-offs in the allocation of resources to maintenance and repair (Kirkwood, 1977; Monaghan et al., 2008). This theory proposes an evolutionary explanation of aging, based on the fact that resources are limited (Kirkwood and Austad, 2000). Organisms need resources for maintenance and repair, but also for other costly functions directly linked to fitness such as reproduction or growth. Because resources are limited, allocation to life history traits involves trade-offs, and selection is expected to favor allocation strategies that maximize fitness (Cole, 1954; Stearns, 1989). Allocating resources to reproduction or growth rather than to maintenance and repair early in life should lead to faster senescence. For example, large breeds of dogs grow faster and exhibit higher mortality than smaller breeds that have not been artificially selected for rapid growth (Kraus et al., 2013). Thus, allocation strategies determine resource availability for different physiological functions and drive cell functioning and deterioration, ultimately affecting life histories including the rate of senescence.

Trade-offs between reproduction and maintenance and the optimal allocation between these traits will depend substantially on resource availability (Baudisch and Vaupel, 2012). For example, female mammals age more rapidly in the wild than in captivity (Lemaître et al., 2013). The consequences of resource limitation, however, can be complex. Dietary restriction can slow senescence in laboratory organisms (Nakagawa et al., 2012). In wild mammals and birds, some vital rates may be sensitive than others, as shown by stronger effects of birth environment on the rates of senescence in survival than in reproduction (Cooper and Kruuk, 2018).

Several studies show that early-life environmental conditions can have long-term effects on senescence in vertebrates (Lindström, 1999; Cooper and Kruuk, 2018; Spagopoulou et al., 2020). For example, poor early environmental conditions were linked with catch-up growth later in life, leading to shorter lifespan in three-spined sticklebacks (*Gasterosteus aculeatus*; Lee et al., 2013, 2016). Female red deer (*Cervus elaphus*) and bighorn sheep (*Ovis canadensis*) born at high population density show, respectively, faster rates of senescence and reduced reproduction and survival compared to those born when intraspecific competition is weak (Nussey et al., 2007; Pigeon et al., 2017). Early-life environment may also have long-term effects on human health (Bateson et al., 2004; Hanson and Gluckman, 2014). These studies emphasized that other environmental factors, including food availability, population density and other stressors, may affect the rate of senescence. Variation in early-life environmental conditions experienced by different cohorts may therefore generate heterogeneity in life-histories and variation in senescence rates between individuals within a population. These differences in life-history patterns reflect underlying trade-offs in soma allocation and are therefore key to better understand both the evolutionary and ecological causes of ageing and its diversity.

Most previous studies on the effect of early environmental conditions on aging considered only one or a few environmental drivers (e.g., Lindström, 1999; Brakefield et al., 2005; Nussey et al., 2007; Pigeon et al., 2017). Studies considering multiple drivers of senescence are scarce and few have investigated of the effects of early-life environment on older age classes in the wild (Rose, 1991; Hammers et al., 2013). Here, we tested whether early environmental conditions, quantified with several environmental variables, or higher reproductive allocation at young ages affected survival and reproduction after the onset of senescence in wild bighorn ewes. We expected that harsh birth environment (high density and high temperature) and high reproductive allocation would lower survival and reproductive success at old age and increase the rates of demographic senescence.

## Materials and Methods

### Study Population

Since 1972, the bighorn sheep population on Ram Mountain (Alberta, Canada, 52° N 115° W, elevation: 1080–2170 m) has been monitored each year between late May and September, the period from birth to weaning of lambs (Jorgenson et al., 1993). Nearly all individuals were marked with colored collars (ewes) or ear tags (lambs and rams). Yearly resighting probability for females was more than 99% (Jorgenson et al., 1997), thus survival was known with precision. Each year, reproduction was monitored by udder inspection at capture and observations of females suckling lambs. We focused on cohorts born between 1973 and 2004, for which all individuals had a known year of death. We restricted analyses to females because males do not provide parental care and their reproductive effort was not quantified. As we were interested in how early environment affected survival and reproductive success at old ages, we considered females past the onset of senescence. We used data for 140 females from 30 cohorts whose age at death ranged from 7 to 19 years (646 female-years) to investigate survival senescence, which begins at age seven in this population (Jorgenson et al., 1997). Female reproductive senescence begins after 11 years of age (Bérubé et al., 1999; Martin and Festa-Bianchet, 2011; Supplementary Figure 1). We therefore restricted analysis of the rate of reproductive senescence to 63 females monitored when 11 years of age or more (205 female-years from 24 cohorts). Females can give birth to one lamb per year. We define successful reproduction as survival of the lamb to late September, the approximate age of weaning (Festa-Bianchet, 1988).

### Early-Life Environment

We examined the effects of weather and population density in the year of birth (Table 1). Data on precipitations (rainfall plus water equivalent of snowfall in mm) and temperature (in °C) were obtained from the Environment Canada meteorological station at Nordegg (52°47′ N, 116°08′ W, elevation: 1,333 m; 25 km from Ram Mountain). These two measures were considered for the winter before birth (early gestation, December to March), spring (late gestation, April and May), summer (birth to weaning, mid-June to September), autumn (mid-September to November) and first winter (December to March). Over these periods, we averaged temperatures and calculated the sum of precipitations. Between 1973 and 2004, temperature increased in summer only (Supplementary Table 1). Precipitation did not show any detectable trends in any season (Supplementary Table 1). Temperature and precipitation affect individual mass changes in this population, making them relevant variables to investigate resource allocation trade-offs (Douhard et al., 2018). We also used the annual mean of the Pacific Decadal Oscillation (PDO), which is negatively related to horn growth (Douhard et al., 2017). This climatic index measures shifts between decades of warm and dry winters, and decades of cooler winters with more precipitation in the Canadian Rocky Mountains (Trenberth and Hurrell, 1994; Whitfield et al., 2010). PDO values were obtained from http://research.jisao.washington.edu/pdo/PDO.latest. Annual PDO did not show any detectable trends over the study period. Population density was estimated by the number of adult females in June each year (Jorgenson et al., 1997). High population density increases competition, and thus corresponds to unfavorable environmental conditions (Nussey et al., 2007; Pigeon et al., 2017). Density peaked at 103 females in 1992 and decreased to a minimum of 18 in 2004. Correlations between environmental variables are in Supplementary Figure 2. Allocation of resources to reproduction is expected to reduce allocation to somatic maintenance and accelerate senescence (Kirkwood and Rose, 1991). We quantified reproductive allocation as the number of lambs weaned prior to the onset of senescence (7 and 11 years of age for survival and reproduction, respectively).

**TABLE 1:** Variables included in models of bighorn sheep female survival and reproductive success at Ram Mountain, Alberta, Canada.

**Variables**	**Description**	**Unit**	**Mean**	**SD**	**Min**	**Max**
Age	Age	years	9.66	2.43	7	19
Spring temperature	Mean temperature during spring preceding birth	°C	3.80	1.46	0.92	6.33
Spring precipitations	Total amount of precipitations during spring preceding birth	mm	104.35	44.03	37.50	229.20
Fall temperature	Mean temperature in autumn following birth	°C	0.08	2.05	−5.33	3.57
Fall precipitations	Total amount of precipitations in autumn following birth	mm	74.77	27.04	30.90	139.80
Summer temperature	Mean temperature during summer of birth	°C	11.19	0.75	9.87	13.08
Summer precipitations	Total amount of precipitations during summer of birth	mm	295.10	96.56	138.40	484.50
Temperature winter before	Mean temperature during winter preceding birth	°C	−7.97	2.43	−12.43	−2.86
Precipitations winter before	Total amount of precipitations during winter preceding birth	mm	84.08	31.23	29.50	158.50
Temperature winter after	Mean temperature during winter following birth	°C	−7.87	2.48	−12.43	−2.86
Precipitations winter after	Total amount of precipitations during winter following birth	mm	84.33	31.23	29.50	158.50
PDO (annual)	Annual mean of the Pacific Decadal Oscillation index	°C	0.32	0.76	−1.10	1.82
Density	Number of females in the population in June		51.81	24.62	18	103
Reproductive allocation	Number of lambs weaned before survival senescence (7 years)		2.61	1.21	0	5
	Number of lambs weaned before reproductive senescence (11 years)		5.64	1.8	1	9

### Statistical Analyses

To investigate the effect of early environment on reproductive success and survival, we performed generalized linear mixed-effects model using the glmer function in the lme4 R package. Survival was a binomial variable representing whether an individual seen in a given year was seen the following year or not. Reproductive success was defined as whether or not a female weaned a lamb in a given year. We quantified the rate of senescence as a linear decline of survival and reproductive success with age after the age of onset. To investigate the effect of environment during early life on senescence rates, we tested the effect of the interaction between age and each early-life environmental variable. Each model therefore included age, one of the environmental variables in early life (temperature and precipitation for each season, PDO, population density, and reproductive allocation) and its interaction with age. Models including only additive effects of age and early-life environment were also tested to investigate if effect of early life persisted at the oldest ages. We report parameter estimates from the additive models when the interaction was non-significant. Environmental variables were tested one at a time to avoid multi-collinearity (Supplementary Figure 4). All environmental variables were centered and scaled. To account for selective disappearance of frail individuals, models for reproduction also included longevity (van de Pol and Verhulst, 2006). We tested the contribution of selective disappearance to rates of senescence by comparing the parameter estimates for age in the best model with and without longevity included (Hayward et al., 2013). Individual identity, cohort and year were also included as random variables.

## Results

**FIGURE 1:**
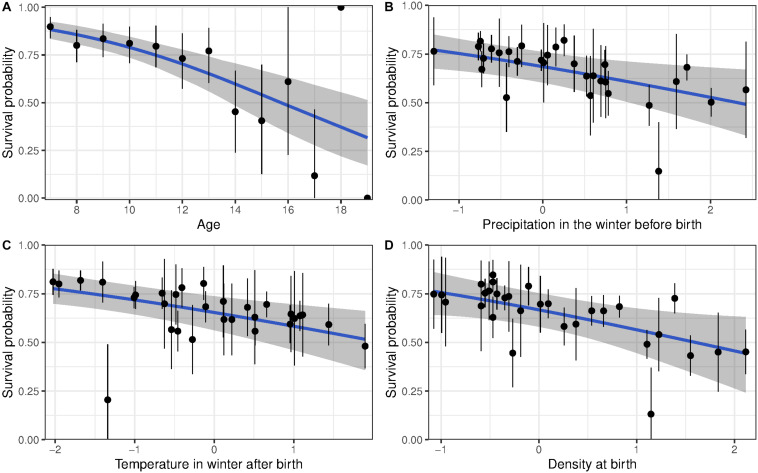
Survival probability of bighorn sheep females born at Ram Mountain, Canada, 1973–2004. **(A)** Shows the age-related decline in survival for females aged 7 years and older, adjusted for current and birth environmental conditions. **(B–D)** show survival of females adjusted to age 13 according to **(B)** precipitation in the winter before birth, **(C)** temperature in the first winter of life, **(D)** population density at birth. The blue line shows predicted survival with associated 95% CI given a linear decline in survival with age. The points in panel **(A)** show average (±95% CI) age-specific survival while points in panels **(B–D)** show each cohort’s survival (±se) adjusted to age 13.

**FIGURE 2:**
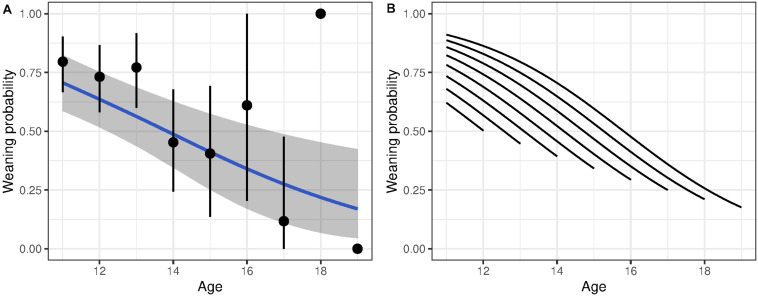
Probability of weaning a lamb as a function of age for bighorn females. **(A)** Shows age-specific probability without accounting for longevity. The blue line shows predicted weaning probability with associated 95% CI given a linear decline in weaning probability with age. The points show average age-specific values (±95% CI). **(B)** Shows the change in weaning success with age decomposed into the effect from within-individual change with age and from selective disappearance. Each line shows the average within-individual change with age of individuals grouped by longevity.

As expected, all models showed a decline in survival and reproductive success with age (Supplementary Tables 2, 3 and Figures 1A, 2A). Early environmental conditions affected reproduction and survival of senescent females (Supplementary Table 2). Heavy precipitations during the winter preceding birth and high temperatures during the winter following birth decreased survival, independently of age (Table 2; Supplementary Table 2, and Figures 1B,C, respectively). Similarly, high population density at birth decreased survival (Table 2 and Figure 1D). However, we found no effect of early-life environment on the rate of actuarial senescence.

**TABLE 2:** Estimated coefficients, standard errors (SE) and corresponding *P*-values of the three generalized linear mixed-effects models showing how survival at old ages is affected by age, precipitations during the winter before preceding birth and temperature during the winter following birth for bighorn sheep females born at Ram Mountain, Canada, from 1973 to 2004, and by population density at birth.

**Model**	**Variables**	**Coefficient**	**SE**	***P*-value**
1	(Intercept)	3.615	0.592	<0.001
	Age	−0.218	0.060	<0.001
	Precipitations (winter before)	−0.334	0.136	0.014
2	(Intercept)	3.485	0.529	<0.001
	Age	−0.219	0.053	<0.001
	Temperature (winter after)	−0.300	0.102	0.003
3	(Intercept)	3.907	0.537	<0.001
	Age	−0.247	0.051	<0.001
	Density	−0.435	0.175	0.013

**FIGURE 3:**
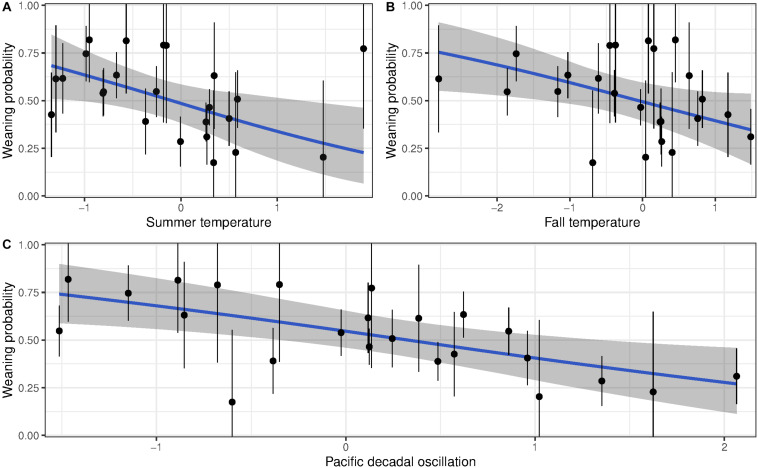
Probability of weaning a lamb for bighorn females born at Ram Mountain, Canada, 1973–2003 according to birth **(A)** summer temperature, **(B)** autumn temperature, and **(C)** Pacific Decadal Oscillation (blue line). Points show the average weaning success (±se) of each cohort adjusted to age 13 years.

For reproductive success, three models showed a detectable effect of early environments (Table 3 and Supplementary Table 3). The variables retained were temperature during summer of birth, temperature the autumn after birth and PDO. Weaning success was 70% for females born in the coldest summers but declined to 25% for those born in the hottest summers (Figure 3A). Similarly, high autumn temperature and PDO in the year of birth decreased late-life reproductive success (Figures 3B,C). Despite the persistent effects of early-life environment on female reproductive success, we found no evidence that it influenced the rate of reproductive senescence (Supplementary Table 3). When selective disappearance was accounted for, the decline in age-specific reproductive success of old females was considerably stronger. Estimates of the rate of reproductive senescence were more pronounced by 30% after accounting for longevity, from −0.35 (95% CI = −0.59, −0.15) to −0.51 [95% CI = −0.78, 0.26 (Figure 2B)]. Selective disappearance likely affects some cohorts more than others, as we found significant relationship between several birth environment variables and longevity (Supplementary Table 4).

**TABLE 3:** Estimated coefficients, standard errors (SE) and corresponding *P*-values of the three generalized linear mixed-effects models showing how reproductive success at old ages is affected by age and by fall temperature, summer temperature and Pacific Decadal Oscillation (PDO) for bighorn sheep females born at Ram Mountain, Canada, from 1973 to 2003.

**Model**	**Variables**	**Coefficient**	**SE**	***P*-value**
1	(Intercept)	2.937	1.477	0.047
	Age	−0.495	0.131	0.000
	Fall temperature	−0.407	0.183	0.026
	Longevity	0.243	0.112	0.029
2	(Intercept)	2.335	1.451	0.108
	Age	−0.486	0.129	0.000
	Summer temperature	−0.612	0.270	0.023
	Longevity	0.274	0.112	0.014
3	(Intercept)	3.295	1.481	0.026
	Age	−0.505	0.133	0.000
	PDO (annual)	−0.569	0.222	0.010
	Longevity	0.241	0.113	0.033

## Discussion

Our results show that early-life environmental conditions of bighorn females correlate with survival and reproductive success late in life. However, early-life environmental conditions influenced survival and reproductive success independently of age for older females. Individuals that experienced high population density at birth, high precipitations during their winter *in utero* and high temperature during their first winter of life had lower survival when aged 7 years and older compared to individuals born under more favorable environmental conditions. In addition, females that experienced high temperature during their first summer and autumn as well as high PDO showed lower reproductive success late in life.

**FIGURE 4:**
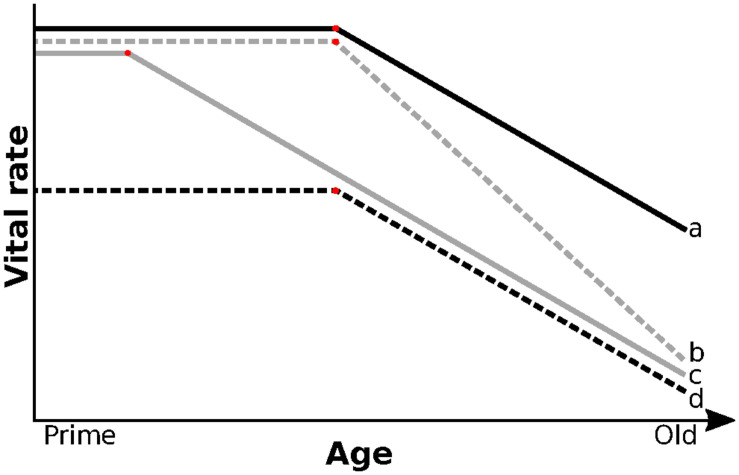
Poor environmental conditions during early life could have different effects on late-life performance. Solid black line (a) illustrates individuals born in good environment. When faced with challenging early life, senescence may show a higher rate (gray dashed: b) or an earlier onset (gray solid, c). Harsh early-life environment may also lower performance independently of age (black dashed; d). In wild populations, patterns of age specific change in performance may be a combination of the above.

Numerous studies reported a link between early-life environment and rates of reproductive but not survival senescence (reviewed by Cooper and Kruuk, 2018). Similarly, we found no evidence that early-life environment affected the rate of survival senescence. However, unlike studies on red deer (Nussey et al., 2007) or red squirrel (*Tamiasciurus hudsonicus*; Descamps et al., 2008), we also found no effect of early-life environment on the rate of reproductive senescence in old females. Bouwhuis et al. (2010), also found that early-life density and food abundance affected the average reproductive success but not the rate of reproductive senescence in great tits (*Parus major*). In Mountain goats (*Oreamnos americanus*), early-life conditions also affected average late-life survival and reproduction without affecting rates of senescence (Panagakis et al., 2017). These mixed results may be due to low sample size at old age, but also to a failure to account for the shape of senescence (Ronget and Gaillard, 2020). Across species, senescence in survival and reproduction show a variety of shapes (Jones et al., 2014; Lemaître et al., 2020), but the impact of early life on the shape of senescence within species remains poorly known. Figure 4 illustrates conceptually how changes in onset and rate of senescence may be shaped by difference in early-life conditions. Compared to individuals born in good conditions (Figure 1A), challenging environmental conditions early in life could increase the rate of senescence or advance its onset (Figure 4b,c, respectively). For example, Hammers et al. (2013) found that early-life environment reduced survival probabilities of Seychelles warblers (*Acrocephalus sechellensis*) independently of age for individuals over 6 years and suggested that this was caused by an earlier onset of senescence (Figure 4c). Alternatively, performance over the entire lifespan may be lower for individuals that develop in poor environments, without changing the rate or onset of senescence (Figure 4d). Individuals may also compensate for a poor start through catch-up growth (Marcil-Ferland et al., 2013) or delayed primiparity (Pigeon and Pelletier, 2018), leading to late-life performances similar to those of individuals that experience favorable early environments (Figure 4a). Distinguishing between these non-mutually exclusive patterns of senescence, however, is impossible if studies focus only on old individuals, which many do, including this study. Non-linear analysis of the entire life of individuals with different early-life environment will be required to disentangle the alternative. That analysis, however, will be challenging because of the small number of individuals per cohort, especially for cohorts born during unfavorable years that have low early survival. Mixture models may provide a solution to this challenge by grouping similar individuals together independently of cohort (Hamel et al., 2017).

The effect of early-life environment on senescence rates may be further obscured by selective disappearance of frail individuals (van de Pol and Verhulst, 2006). In bighorn females, large body mass in prime age is associated with greater longevity (Bérubé et al., 1999) and mass is influenced by early-life environment (Pigeon and Pelletier, 2018). Frail females could also die as young adults and only those robust enough to withstand the negative effect of high precipitations in the year of birth may survive to older ages. Even among older females, selective disappearance plays an important role, decreasing the apparent rates of senescence by 30%. That disappearance is not random in relation to birth environment, as individuals born in harsh environments (Supplementary Table 4) die at an earlier age. The effects of selective disappearance on vital rates are of similar magnitude to those reported for Soay sheep (*Ovis aries*; Hayward et al., 2013). Despite its importance, this selective effect is probably under-estimated in our study because only 14% of females survive from the time they are marked to the onset of reproductive senescence at 11 years (Supplementary Figure 3). Selection against frail individuals early in life is therefore most likely excluded from our estimate.

Our study suggests that a poor start can leave a permanent signature on fitness despite large variation in subsequent environmental conditions (Pigeon and Pelletier, 2018), although we did not investigate the mechanisms behind these patterns. Rapid development early in life, including catch-up growth (Marcil-Ferland et al., 2013), often has negative long-term effects. Several physiological mechanisms causing long-term effects have been proposed, including epigenetic programming (Heijmans et al., 2008; Bar-Sadeh et al., 2020). Stress during early life has also been associated with shorter telomeres and mitochondrial inefficiency (Casagrande et al., 2020; but see Brown et al., 2021). Drosophila (*Drosophila melanogaster*) reared on a poor diet show a higher rate of germline stem cell decline due to changes in insulin signaling (Hsu and Drummond-Barbosa, 2009), which is linked to reproductive senescence (Quesada-Candela et al., 2021). Linking physiological process with late-life fitness in natural conditions will be critical to understand the mechanisms of senescence.

The disposable soma hypothesis predicts that allocation of resources to reproduction rather than to somatic maintenance should lead to faster senescence (Kirkwood and Rose, 1991). Detection of such tradeoffs is common, but not universal (Lemaître et al., 2015). Unlike red deer (Nussey et al., 2007) and common guillemots (*Uria aalge*; Reed et al., 2008), in bighorn sheep we found no evidence that increased reproductive allocation decreases survival of old females or increases their rate of actuarial senescence. On the contrary, we found a trend for positive covariance between early reproductive allocation and late-life survival, confirming an earlier (Bérubé et al., 1999). This result is consistent with studies of other long-lived capital breeders like elephant seals (*Mirounga leonina*; Oosthuizen et al., 2021) and mountain goats (Panagakis et al., 2017) that did not detect early-late life trade-offs. Strong individual heterogeneity can limit our ability to detect life-history trade-offs through phenotypic correlations (Hamel et al., 2010). The exact source of this heterogeneity, however, remains to be explored and may be unrelated to early-life environment. While the effect of early-life environmental conditions on allocation trade-offs and their consequence on senescence has received much attention (Lemaître et al., 2015), fewer studies have considered allocation throughout the lifespan. In long-lived species, events occurring at maturity may also play a more important role (Baudisch and Vaupel, 2012). Heterogeneity may also stem from factors other than resource scarcity. For example, in baboons (*Papio spp.*), early social environment, which varies greatly between individuals, plays a predominant role (Alberts, 2019). Thus, variation in social context experienced by different cohorts or individuals can, generates heterogeneity in life-history, even within a single population. Future studies should therefore consider a broader view of the drivers of variation in senescence patterns, and not only early-life resource scarcity. Since senescence is the result of resource allocation decisions made to face stressors through life, individual experiences, at birth but also later in life, may be just as important.

## Data Availability Statement

The raw data supporting the conclusions of this article will be made available by the authors, without undue reservation.

## Ethics Statement

The animal study was reviewed and approved by Animal-handling procedures were approved by the Animal Care Committee of the University of Sherbrooke (protocol 2020-2707).

## Author Contributions

GP and JL performed the analyses. GP, JL, MF-B, and FP contributed to the data collection, conception, and writing of the manuscript. All authors contributed to the article and approved the submitted version.

## Conflict of Interest

The authors declare that the research was conducted in the absence of any commercial or financial relationships that could be construed as a potential conflict of interest.
