# Supplementary material for: Do Early-Life Conditions Drive Variation in Senescence of Female Bighorn Sheep?
Source: Front Cell Dev Biol. 2021 May 20;9:637692. doi: 10.3389/fcell.2021.637692 (PMC8173223; doi:10.3389/fcell.2021.637692)
Supplement: Supplementary file 1 [file Data_Sheet_1.docx]

**SUPPORTING INFORMATION**

**Supplementary Tables**

**Table S1.**  Estimated temporal trend (change per year) of environmental variables from 1973 to 2004 at Ram Mountain, based on linear regression models.

| **Variable** | **Season** | **Beta** | **SE** | **P** |
| --- | --- | --- | --- | --- |
| Temperature | Spring | -0.006 | 0.028 | 0.827 |
|  | Summer | 0.029 | 0.014 | **0.038** |
|  | Fall | 0.027 | 0.04 | 0.495 |
|  | Winter | 0.035 | 0.047 | 0.454 |
| Precipitation | Spring | -0.689 | 0.848 | 0.422 |
|  | Summer | -0.102 | 1.88 | 0.957 |
|  | Fall | 0.186 | 0.525 | 0.726 |
|  | Winter | -0.528 | 0.6 | 0.386 |
| PDO | | 0.007 | 0.015 | 0.644 |
| Density | | 0.504 | 0.47 | 0.292 |

**Table S2**. Estimated coefficients, standard errors (SE) and the corresponding p-values of generalized linear mixed-effects models showing the effect of age and early-life environmental conditions on survival at old ages.

|  | **Interactions** | | | **Additive** | | |
| --- | --- | --- | --- | --- | --- | --- |
| **Variables** | **Coef.** | **SE** | **P-value** | **Coef.** | **SE** | **P-value** |
| (Intercept) | 3.726 | 0.569 | <0.001 | 3.643 | 0.569 | <0.001 |
| Age | -0.241 | 0.057 | <0.001 | -0.232 | 0.057 | <0.001 |
| Temperature (spring) | 0.344 | 0.417 | 0.409 | -0.009 | 0.125 | 0.944 |
| Age: Temperature (spring) | -0.036 | 0.041 | 0.375 | - | - | - |
| (Intercept) | 3.730 | 0.571 | <0.001 | 3.648 | 0.526 | <0.001 |
| Age | -0.250 | 0.058 | <0.001 | -0.241 | 0.053 | <0.001 |
| Precipitation (spring) | 0.112 | 0.440 | 0.800 | 0.267 | 0.144 | 0.062 |
| Age : Precipitation (spring) | 0.015 | 0.040 | 0.708 | - | - | - |
| (Intercept) | 3.737 | 0.565 | <0.001 | 3.643 | 0.569 | <0.001 |
| Age | -0.241 | 0.056 | <0.001 | -0.231 | 0.057 | <0.001 |
| Temperature (fall) | 0.477 | 0.482 | 0.323 | 0.036 | 0.136 | 0.791 |
| Age: Temperature (fall) | -0.045 | 0.047 | 0.343 | - | - | - |
| (Intercept) | 3.602 | 0.564 | <0.001 | 3.721 | 0.572 | <0.001 |
| Age | -0.222 | 0.055 | <0.001 | -0.231 | 0.056 | <0.001 |
| Precipitation (fall) | 0.507 | 0.489 | 0.300 | -0.228 | 0.130 | 0.079 |
| Age : Precipitation (fall) | -0.075 | 0.048 | 0.121 | - | - | - |
| (Intercept) | 3.721 | 0.610 | <0.001 | 3.557 | 0.589 | <0.001 |
| Age | -0.235 | 0.063 | <0.001 | -0.218 | 0.061 | <0.001 |
| Temperature (summer) | 0.827 | 0.694 | 0.233 | 0.158 | 0.199 | 0.429 |
| Age : Temperature (summer) | -0.068 | 0.068 | 0.314 | - | - | - |
| (Intercept) | 3.355 | 0.601 | <0.001 | 3.624 | 0.571 | <0.001 |
| Age | -0.205 | 0.060 | 0.001 | -0.231 | 0.057 | <0.001 |
| Precipitation (summer) | 0.607 | 0.525 | 0.248 | 0.030 | 0.161 | 0.854 |
| Age : Precipitation (summer) | -0.054 | 0.047 | 0.250 | - | - | - |
| (Intercept) | 3.779 | 0.607 | <0.001 | 3.639 | 0.567 | <0.001 |
| Age | -0.246 | 0.061 | <0.001 | -0.231 | 0.057 | <0.001 |
| Temperature (winter before) | 0.360 | 0.491 | 0.464 | 0.012 | 0.153 | 0.936 |
| Age : Temperature (winter before) | -0.034 | 0.045 | 0.456 | - | - | - |
| (Intercept) | 3.625 | 0.598 | <0.001 | 3.615 | 0.592 | <0.001 |
| Age | -0.218 | 0.060 | <0.001 | -0.218 | 0.060 | <0.001 |
| Precipitation (winter before) | -0.500 | 0.472 | 0.289 | -0.334 | 0.136 | 0.014 |
| Age : Precipitation (winter before) | 0.017 | 0.047 | 0.713 | - | - | - |
| (Intercept) | 3.712 | 0.595 | <0.001 | 3.485 | 0.529 | <0.001 |
| Age | -0.245 | 0.061 | <0.001 | -0.219 | 0.053 | <0.001 |
| Temperature (winter after) | 0.069 | 0.418 | 0.869 | -0.300 | 0.102 | 0.003 |
| Age : Temperature (winter after) | -0.037 | 0.041 | 0.363 | - | - | - |
| (Intercept) | 3.528 | 0.600 | <0.001 | 3.581 | 0.562 | <0.001 |
| Age | -0.226 | 0.060 | <0.001 | -0.231 | 0.057 | <0.001 |
| Precipitation (winter after) | 0.258 | 0.513 | 0.616 | 0.136 | 0.151 | 0.367 |
| Age : Precipitation (winter after) | -0.011 | 0.046 | 0.804 | - | - | - |
| (Intercept) | 3.739 | 0.582 | <0.001 | 3.773 | 0.566 | <0.001 |
| Age | -0.239 | 0.056 | <0.001 | -0.242 | 0.055 | <0.001 |
| PDO (annual) | -0.060 | 0.590 | 0.919 | -0.185 | 0.176 | 0.292 |
| Age : PDO (annual) | -0.012 | 0.054 | 0.825 | - | - | - |
| (Intercept) | 3.893 | 0.543 | <0.001 | 3.907 | 0.537 | <0.001 |
| Age | -0.246 | 0.052 | <0.001 | -0.247 | 0.051 | <0.001 |
| Density | -0.352 | 0.572 | 0.538 | -0.435 | 0.175 | 0.013 |
| Age : Density | -0.009 | 0.057 | 0.879 | - | - | - |
| (Intercept) | 2.693 | 1.123 | 0.016 | 3.228 | 0.555 | <0.001 |
| Age | -0.188 | 0.113 | 0.096 | -0.242 | 0.053 | <0.001 |
| Reproductive allocation | 0.391 | 0.374 | 0.295 | 0.196 | 0.105 | 0.060 |
| Age : Reproductive allocation | -0.019 | 0.035 | 0.587 | - | - | - |

**Table S3**. Estimated coefficients, standard errors (SE) and the corresponding p-values of generalized linear mixed-effects models showing the effect of age and early-life environmental conditions on reproductive success at old ages.

|  | **Interactions** | | | **Additive** | | |
| --- | --- | --- | --- | --- | --- | --- |
| **Variables** | **Coef.** | **SE** | **P-value** | **Coef.** | **SE** | **P-value** |
| (Intercept) | 2.873 | 1.522 | 0.059 | 2.666 | 1.466 | 0.069 |
| Age | -0.509 | 0.142 | 3.28e-04 | -0.482 | 0.134 | 3.19e-04 |
| Temperature (spring) | 0.784 | 1.431 | 0.584 | -0.100 | 0.159 | 0.528 |
| Longevity | 0.266 | 0.117 | 0.023 | 0.258 | 0.117 | 0.027 |
| Age: Temperature (spring) | -0.072 | 0.116 | 0.534 | - | - | - |
| (Intercept) | 2.977 | 1.888 | 0.115 | 2.810 | 1.509 | 0.063 |
| Age | -0.496 | 0.157 | 0.002 | -0.484 | 0.135 | 3.24e-04 |
| Precipitation (spring) | -0.096 | 1.226 | 0.938 | 0.084 | 0.183 | 0.645 |
| Longevity | 0.248 | 0.121 | 0.040 | 0.249 | 0.120 | 0.038 |
| Age : Precipitation (spring) | 0.014 | 0.093 | 0.882 | - | - | - |
| (Intercept) | 3.397 | 1.615 | 0.036 | 2.937 | 1.477 | 0.047 |
| Age | -0.543 | 0.147 | 2.12e-04 | -0.495 | 0.131 | 1.51e-04 |
| Temperature (fall) | 0.945 | 1.722 | 0.583 | -0.407 | 0.183 | 0.026 |
| Longevity | 0.251 | 0.112 | 0.025 | 0.243 | 0.112 | 0.029 |
| Age: Temperature (fall) | -0.107 | 0.136 | 0.431 | - | - | - |
| (Intercept) | 2.742 | 1.575 | 0.082 | 2.784 | 1.575 | 0.077 |
| Age | -0.477 | 0.136 | 4.55e-04 | -0.485 | 0.135 | 3.38e-04 |
| Precipitation (fall) | -0.798 | 1.547 | 0.606 | -0.045 | 0.200 | 0.823 |
| Longevity | 0.252 | 0.121 | 0.037 | 0.255 | 0.121 | 0.035 |
| Age : Precipitation (fall) | 0.061 | 0.125 | 0.623 | - | - | - |
| (Intercept) | 3.157 | 1.585 | 0.046 | 2.335 | 1.451 | 0.108 |
| Age | -0.576 | 0.146 | 7.72e-05 | -0.486 | 0.129 | 1.64e-04 |
| Temperature (summer) | 3.157 | 2.224 | 0.156 | -0.612 | 0.270 | 0.023 |
| Longevity | 0.294 | 0.113 | 0.009 | 0.274 | 0.112 | 0.014 |
| Age : Temperature (summer) | -0.301 | 0.177 | 0.089 | - | - | - |
| (Intercept) | 3.253 | 1.654 | 0.049 | 2.757 | 1.511 | 0.068 |
| Age | -0.524 | 0.145 | 3.02e-04 | -0.486 | 0.135 | 3.35e-04 |
| Precipitation (summer) | -1.202 | 1.403 | 0.392 | -0.064 | 0.183 | 0.728 |
| Longevity | 0.259 | 0.119 | 0.029 | 0.260 | 0.118 | 0.028 |
| Age : Precipitation (summer) | 0.088 | 0.108 | 0.413 | - | - | - |
| (Intercept) | 2.108 | 1.560 | 0.177 | 2.656 | 1.484 | 0.074 |
| Age | -0.436 | 0.142 | 0.002 | -0.485 | 0.135 | 3.14e-04 |
| Temperature (winter before) | -1.303 | 1.337 | 0.330 | -0.074 | 0.183 | 0.684 |
| Longevity | 0.258 | 0.115 | 0.025 | 0.262 | 0.118 | 0.026 |
| Age : Temperature (winter before) | 0.098 | 0.105 | 0.352 | - | - | - |
| (Intercept) | 2.399 | 1.549 | 0.121 | 2.513 | 1.532 | 0.101 |
| Age | -0.476 | 0.136 | 4.50e-04 | -0.481 | 0.135 | 3.61e-04 |
| Precipitation (winter before) | -0.890 | 1.752 | 0.612 | 0.079 | 0.206 | 0.703 |
| Longevity | 0.274 | 0.120 | 0.023 | 0.270 | 0.120 | 0.025 |
| Age : Precipitation (winter before) | 0.077 | 0.139 | 0.578 | - | - | - |
| (Intercept) | 2.766 | 1.908 | 0.147 | 2.822 | 1.559 | 0.070 |
| Age | -0.479 | 0.164 | 0.004 | -0.484 | 0.135 | 3.41e-04 |
| Temperature (winter after) | -0.122 | 1.343 | 0.928 | -0.054 | 0.171 | 0.751 |
| Longevity | 0.249 | 0.123 | 0.043 | 0.249 | 0.123 | 0.043 |
| Age : Temperature (winter after) | 0.005 | 0.107 | 0.960 | - | - | - |
| (Intercept) | 3.088 | 1.570 | 0.049 | 2.736 | 1.465 | 0.062 |
| Age | -0.533 | 0.153 | 4.76e-04 | -0.484 | 0.132 | 2.45e-04 |
| Precipitation (winter after) | -0.824 | 1.409 | 0.559 | 0.163 | 0.172 | 0.344 |
| Longevity | 0.268 | 0.117 | 0.023 | 0.251 | 0.114 | 0.027 |
| Age : Precipitation (winter after) | 0.077 | 0.110 | 0.481 | - | - | - |
| (Intercept) | 3.410 | 1.468 | 0.020 | 3.295 | 1.481 | 0.026 |
| Age | -0.508 | 0.130 | 9.82e-05 | -0.505 | 0.133 | 1.39e-04 |
| PDO (annual) | -1.871 | 1.567 | 0.232 | -0.569 | 0.222 | 0.010 |
| Longevity | 0.238 | 0.112 | 0.033 | 0.241 | 0.113 | 0.033 |
| Age : PDO (annual) | 0.102 | 0.121 | 0.398 | - | - | - |
| (Intercept) | 2.579 | 1.495 | 0.084 | 2.654 | 1.501 | 0.077 |
| Age | -0.480 | 0.135 | 3.64e-04 | -0.483 | 0.135 | 3.48e-04 |
| Density | -0.972 | 1.866 | 0.602 | 0.020 | 0.293 | 0.946 |
| Longevity | 0.265 | 0.118 | 0.024 | 0.262 | 0.119 | 0.027 |
| Age : Density | 0.078 | 0.145 | 0.591 | - | - | - |
| (Intercept) | -5.707 | 4.703 | 0.225 | 2.005 | 1.487 | 0.178 |
| Age | 0.103 | 0.367 | 0.779 | -0.494 | 0.135 | 2.61e-04 |
| Reproductive allocation | 1.476 | 0.767 | 0.054 | 0.192 | 0.102 | 0.060 |
| Longevity | 0.252 | 0.113 | 0.025 | 0.247 | 0.116 | 0.037 |
| Age : Reproductive allocation | -0.101 | 0.059 | 0.089 | - | - | - |

**Table S4:** effect of early-life environment and reproductive allocation on longevity for the 205 female ewes that lived past the onset of reproductive senescence. Coefficients were obtained from a linear mixed model of longevity as a function of birth environment, with cohort as a random effect.

| **Variables** | **Coef.** | **SE** | **95% CI** | |
| --- | --- | --- | --- | --- |
| Temperature (spring) | -0.26 | 0.32 | -0.88 | 0.36 |
| Precipitation (spring) | 0.57 | 0.33 | -0.07 | 1.21 |
| Temperature (fall) | -0.35 | 0.34 | -1.01 | 0.32 |
| **Precipitation (fall)** | **-0.7** | **0.3** | **-1.29** | **-0.11** |
| Temperature (summer) | -0.69 | 0.45 | -1.57 | 0.18 |
| Precipitation (summer) | -0.24 | 0.4 | -1.01 | 0.54 |
| Temperature (winter before) | -0.21 | 0.38 | -0.95 | 0.55 |
| Precipitation (winter before) | -0.62 | 0.42 | -1.46 | 0.21 |
| **Temperature (winter after)** | **-0.81** | **0.28** | **-1.37** | **-0.27** |
| Precipitation (winter after) | 0.38 | 0.36 | -0.32 | 1.09 |
| PDO (annual) | -0.39 | 0.38 | -1.12 | 0.35 |
| Density | -0.75 | 0.41 | -1.57 | 0.06 |
| Reproductive allocation | 0.19 | 0.14 | -0.09 | 0.47 |

**Supplementary Figures**


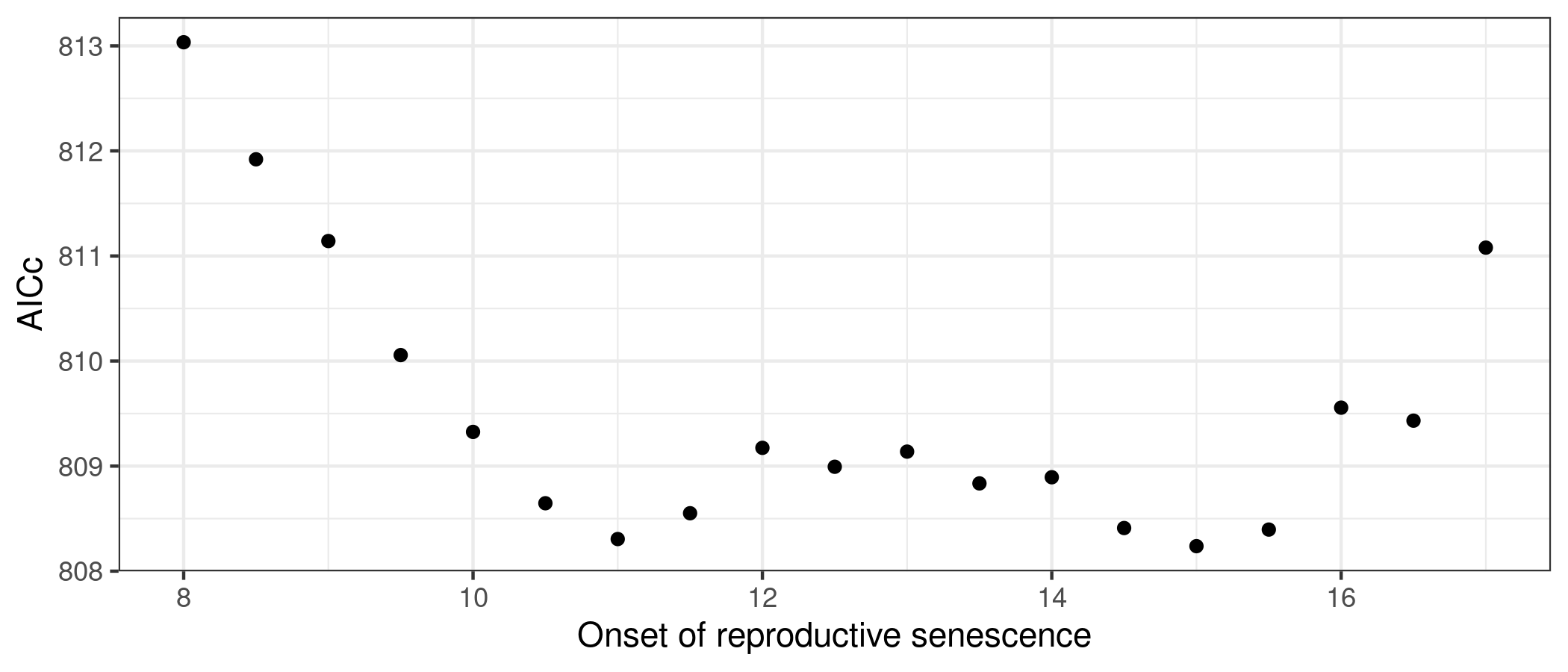
**Figure S1:** AICc selection to determine the onset of reproductive senescence. Weaning probability of big horn ewes of 7 years and more was fitted as a function of age using a broken-stick model with different thresholds for the onset of senescence ranging from 8 to 17 years of age. The models included longevity as a control variable as well as year, id, and cohort as random effects.


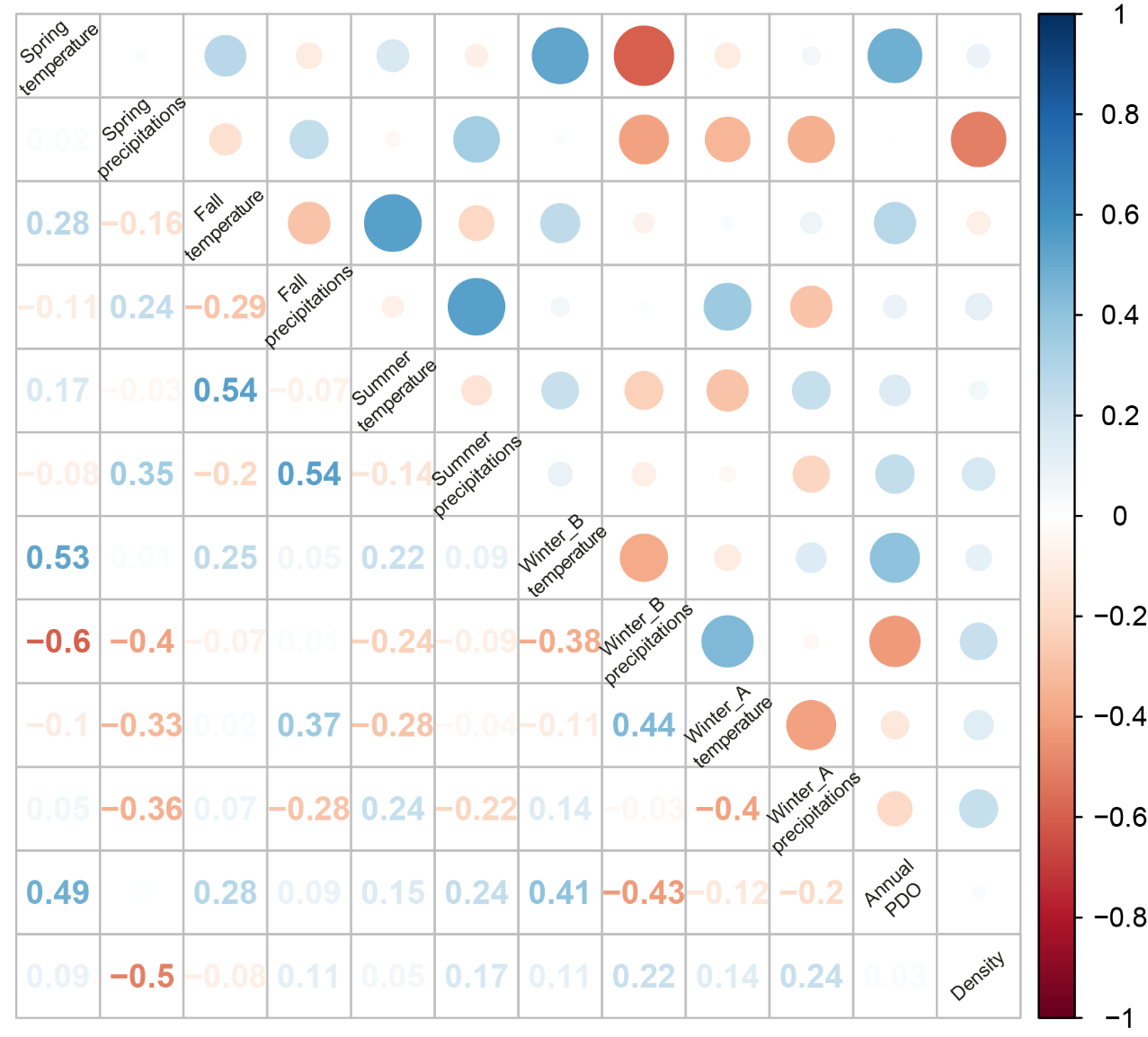


**Figure S2**. Correlation matrices of centered and scaled variables describing early-life environment. Lower panel: Correlation coefficients. Upper panel: Correlogram. Circle size is proportional to the corresponding correlation coefficient. Positive and negative correlations are represented respectively in red and blue.


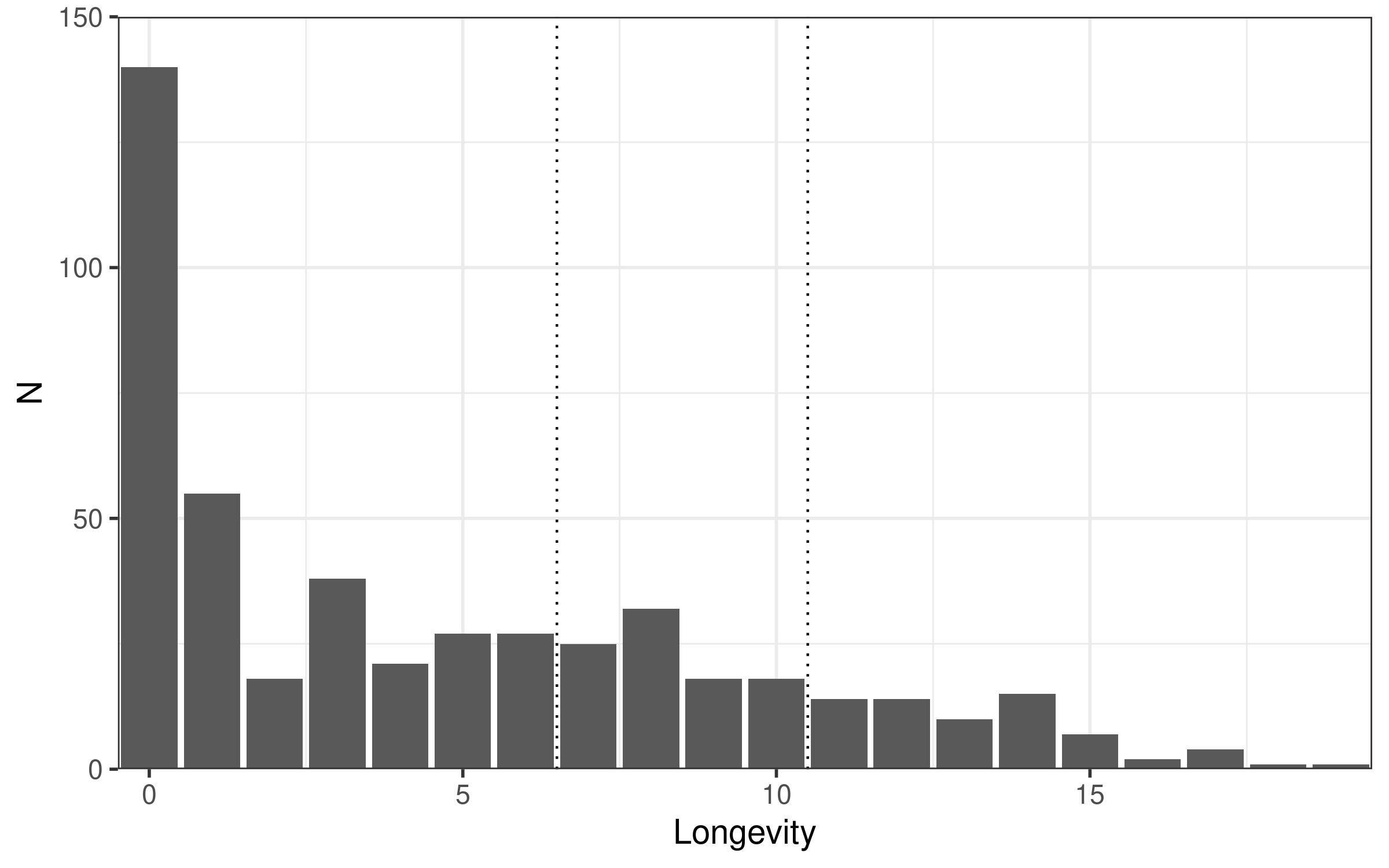
**Figure S3:** Distribution of longevity for bighorn sheep ewes of Ram Mountain born between 1973 and 2004. Vertical dotted lines at 7 and 11 show the onset of actuarial and reproductive senescence, respectively.
